# Linking protein to phenotype with Mendelian Randomization detects 38 proteins with causal roles in human diseases and traits

**DOI:** 10.1371/journal.pgen.1008785

**Published:** 2020-07-06

**Authors:** Andrew D. Bretherick, Oriol Canela-Xandri, Peter K. Joshi, David W. Clark, Konrad Rawlik, Thibaud S. Boutin, Yanni Zeng, Carmen Amador, Pau Navarro, Igor Rudan, Alan F. Wright, Harry Campbell, Veronique Vitart, Caroline Hayward, James F. Wilson, Albert Tenesa, Chris P. Ponting, J. Kenneth Baillie, Chris Haley

**Affiliations:** 1 MRC Human Genetics Unit, Institute of Genetics and Molecular Medicine, University of Edinburgh, Western General Hospital, Edinburgh, Scotland, United Kingdom; 2 The Roslin Institute, University of Edinburgh, Easter Bush, Edinburgh, Scotland, United Kingdom; 3 Centre for Global Health Research, Usher Institute, University of Edinburgh, Teviot Place, Edinburgh, Scotland, United Kingdom; 4 Faculty of Forensic Medicine, Zhongshan School of Medicine, Sun Yat-Sen University, Guangzhou, China; 5 Guangdong Province Translational Forensic Medicine Engineering Technology Research Center, Zhongshan School of Medicine, Sun Yat-Sen University, Guangzhou, China; 6 Guangdong Province Key Laboratory of Brain Function and Disease, Zhongshan School of Medicine, Sun Yat-Sen University, Guangzhou, China; University of Bristol, UNITED KINGDOM

## Abstract

To efficiently transform genetic associations into drug targets requires evidence that a particular gene, and its encoded protein, contribute causally to a disease. To achieve this, we employ a three-step proteome-by-phenome Mendelian Randomization (MR) approach. In step one, 154 protein quantitative trait loci (pQTLs) were identified and independently replicated. From these pQTLs, 64 replicated locally-acting variants were used as instrumental variables for proteome-by-phenome MR across 846 traits (step two). When its assumptions are met, proteome-by-phenome MR, is equivalent to simultaneously running many randomized controlled trials. Step 2 yielded 38 proteins that significantly predicted variation in traits and diseases in 509 instances. Step 3 revealed that amongst the 271 instances from GeneAtlas (UK Biobank), 77 showed little evidence of pleiotropy (HEIDI), and 92 evidence of colocalization (eCAVIAR). Results were wide ranging: including, for example, new evidence for a causal role of tyrosine-protein phosphatase non-receptor type substrate 1 (SHPS1; *SIRPA*) in schizophrenia, and a new finding that intestinal fatty acid binding protein (FABP2) abundance contributes to the pathogenesis of cardiovascular disease. We also demonstrated confirmatory evidence for the causal role of four further proteins (FGF5, IL6R, LPL, LTA) in cardiovascular disease risk.

## Author summary

The targets of most medications prescribed today are proteins. For many common diseases our understanding of the underlying causes is often incomplete, and our ability to predict whether new drugs will be effective is remarkably poor. Attempts to use genetics to identify drug targets have an important limitation: standard study designs link disease risk to DNA but do not explain how the genotype leads to disease. In our study, we made robust statistical links between DNA variants and blood levels of 249 proteins, in two separate groups of Europeans. We then used this information to predict protein levels in large genetic studies. In many cases, this second step gives us evidence that high or low levels of a given protein play a role in causing a given disease. Among dozens of high-confidence links, we found new evidence for a causal role of a protein called SHPS1 in schizophrenia, and of another protein (FABP2) in heart disease. Our method takes advantage of information from large numbers of existing genetic studies to prioritize specific proteins as drug targets.

## Introduction

An initial goal of drug development is the identification of targets—in most cases, proteins—whose interaction with a drug ameliorates the development, progression, or symptoms of disease. After some success, the rate of discovery of new targets has not accelerated despite substantially increased investment [1]. A large proportion of drugs fail during the last stages of development—clinical trials—because their targets do not alter whole-organism phenotypes as expected from observational and other pre-clinical research [2]. Genetic approaches to drug development [3] offer a distinct advantage over observational studies. It is estimated that by selecting targets with genetic evidence, the chance of success of those targets doubles in subsequent clinical development [4]. For example, a recent study found that 12% of all targets for licensed drugs could be rediscovered using GWA studies [5]. Indeed, there have been a number of recent high-profile successes prioritizing therapeutic targets at genome-wide scales [6,7]. Nevertheless, the genetic associations of disease are often still not immediately interpretable [8] and many disease-associated variants alter protein levels via poorly understood mechanisms.

When combined with proteomic data, however, genetics can provide insight into proteins that likely impact disease pathogenesis. Mendelian Randomization (MR) in this context uses genetic variants to estimate the effect of an exposure on an outcome, using the randomness by which alleles are allocated to gametes to remove the effects of unmeasured confounding between a protein and the outcome [9]. Given a set of assumptions, detailed below, this approach is analogous to a naturally-occurring randomized controlled trial. Using a genetic variant that predicts the abundance of a mediating molecule, MR tests the hypothesis that this molecule plays a causal role in disease risk. To do so it takes advantage of the patient’s, or participant’s, randomization at conception to this molecule’s genetically-determined level. Under this model, it is possible to use population level genetic information to draw causal inference from observational data.

Proteome-by-phenome MR, in common with all other MR studies, has three key assumptions that must be fulfilled to ensure the legitimacy of any causal conclusions drawn [10]: 1) that the SNP is associated with the exposure of interest, 2) that the SNP is independent of any confounders, and 3) that the SNP does not influence the outcome of interest, except via the exposure variable.

A common concern in the use of MR is that the genetic variant is linked to the outcome phenotype via an alternative causal pathway. In a drug trial this would be analogous to an intervention influencing a clinical outcome through a different pathway than via its reported target. To avoid pursuing drugs that target an irrelevant molecular entity, and hence that have no beneficial effect, we applied MR to proteins—the likely targets of therapy—and limited our genetic variants to those that are locally-acting protein quantitative trait loci (pQTLs). This approach provides stronger supporting evidence for a causal role of the protein on disease than relying on the proximity of a disease-associated genetic variant to a nearby gene, or using mRNA abundance as a proxy for protein abundance [11].

Previous studies have also leveraged the increased availability of pQTL data for drug target and biomarker discovery [12–18]. For example, in one of the largest pQTL studies to date, Sun et al. [14] applied an aptamer-based approach (rather than an antibody-based assay as here) to perform extensive co-localization analyses and used MR to assess the causal contribution of IL1RL1–IL18R1 locus to atopic dermatitis, and that of MMP12 to coronary heart disease. In the study presented here, we attempt to systematically use MR to link protein to outcome trait by taking a three-step approach. Firstly, identifying replicated pQTL in our two European cohort studies before then using these in a systematic MR approach with two large sets of GWA study data. In a final step, we test results from one of these sets for evidence of heterogeneity and colocalization of effects.

Overall, our proteome-by-phenome MR approach assessed the causal role of 64 proteins in 846 outcomes (e.g. diseases, anthropomorphic measures, etc.), identifying 38 as causally contributing to human diseases or other quantitative traits. Notwithstanding the assumptions of MR, obtaining evidence for causality from studies such as this is far more scalable than via randomized controlled trials, and is more physiologically relevant than model organism studies.

## Results

### Protein QTLs

The abundance of an individual protein can be associated with DNA variants that are either local or distant to its gene (termed local- and distal-pQTLs, respectively). In many respects, locally-acting pQTLs are ideal instrumental variables for MR: they tend to have large effect sizes, have highly plausible biological relationships with protein level, and provide quantitative information about (often) directly druggable protein targets. This is in contrast to distal pQTLs, where the pathway through which they exert their effects is generally unknown, with no *a priori* expectation of a direct effect on a single target gene.

We assayed the plasma levels of 249 proteins using high-throughput, multiplex immunoassays and then performed genome-wide association of these levels in each of two independent cohorts (discovery and replication) of 909 and 998 European individuals who had previously been genotyped.

**Fig 1:**
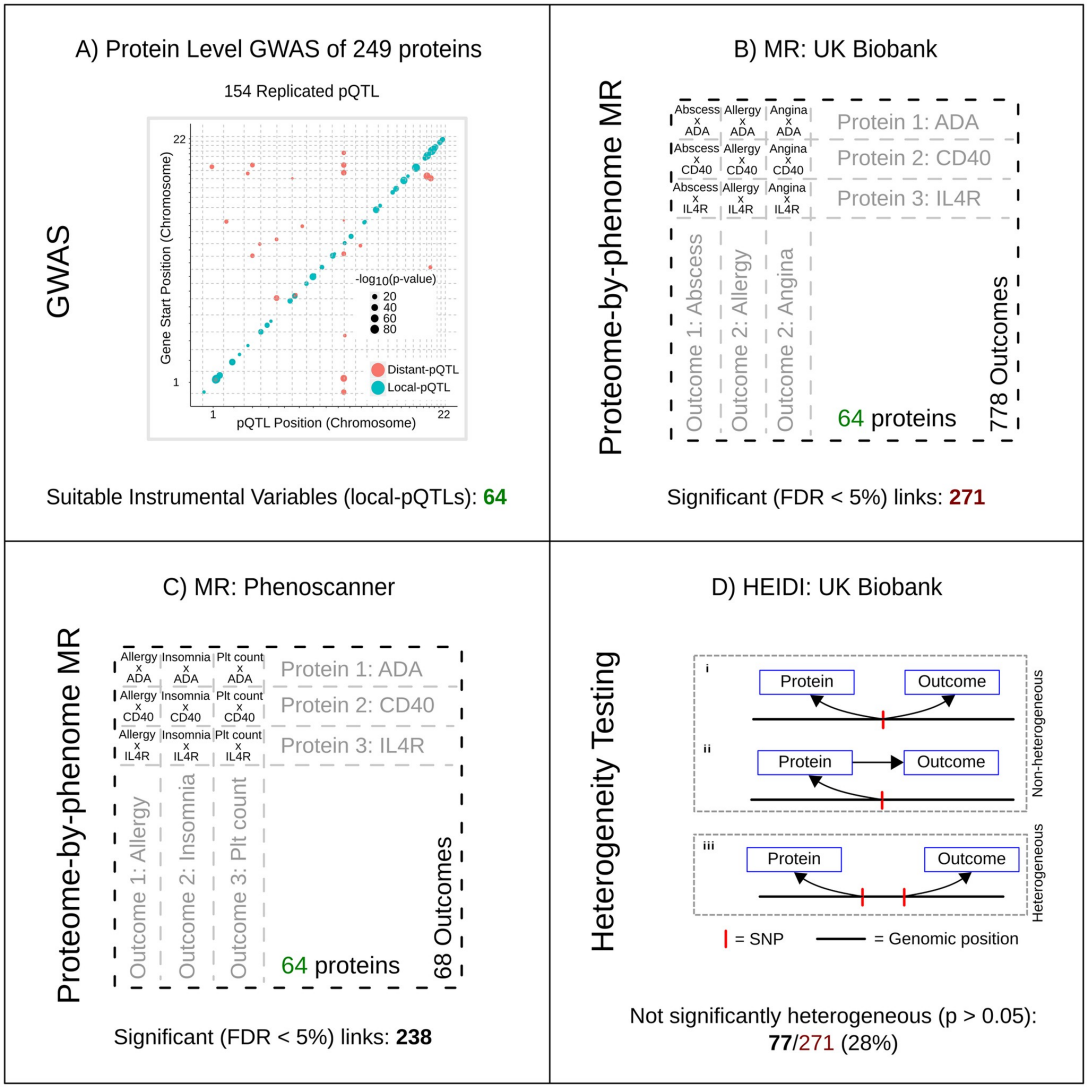
Proteome-by-phenome Mendelian Randomization. A) Genome-wide associations of the plasma concentrations of 249 proteins from two independent European cohorts (discovery and replication) were calculated. The plot shows pQTL position against chromosomal location of the gene that encodes the protein under study for all replicated pQTLs. The area of a filled circle is proportional to its -log10(p-value) in the replication cohort. Blue circles indicate pQTLs ±150kb of the gene (‘local-pQTLs’); red circles indicate pQTLs more than 150kb from the gene. B, C) Local-pQTLs of 64 proteins were taken forward for proteome-by-phenome MR analysis. These were assessed against 778 outcome phenotypes from GeneAtlas [20] (panel B; UK Biobank) and 68 phenotypes identified using Phenoscanner [21,22] (panel C). In each set of results an FDR of <0.05 was considered significant. D) Heterogeneity in dependent instruments (HEIDI [23]) testing was undertaken for MR significant results from GeneAtlas (n = 271). This test seeks to distinguish a single causal variant at a locus effecting both exposure and outcome directly (as in i) or in a causal chain (as in ii), from two causal variants in linkage disequilibrium (as in iii), one affecting the exposure and the other effecting the outcome.

Lead-SNPs, defined as the variant with the smallest p-value and accounting for linkage disequilibrium (Methods), were identified for each protein. As expected, pQTLs were highly concordant between the two independent cohorts (S1 Table). 121 pQTL were identified in the discovery dataset, and, of these, 90.1% (109/121) were successfully replicated after accounting for multiple testing in both the discovery and replication. However, this was felt to be excessively stringent with respect to instrument identification, and a more permissive threshold of 5x10^-8^ was therefore used in the discovery cohort. Of the 209 lead-SNPs identified in the discovery cohort at this threshold, 154 were successfully replicated (accounting for multiple testing during replication and with consistent direction of effect). These represented pQTLs for 82 proteins, all but two proteins were successfully mapped to an autosomal gene (Ensembl GRCh37). The majority of these proteins (64/80; 80%) had a replicated lead-SNP within 150kb of the gene encoding the protein (Fig 1). The variant to use as the instrumental variable for each protein was selected as the replicated lead-SNP lying within 150kb of the gene encoding the protein with the lowest significant p-value in the discovery set (Methods). Increasing this proximity threshold to within 1Mb added a single protein only. Further support for the validity of these instruments was provided through comparison with the results of Sun et al. [14] and GTEx [19] (Methods): of the instrumental variables identified (a) 52% (14/27) of those comparable were in high LD (r^2^>0.8) with the results of Sun et al. (S2 Table), and (b) 30% (16/54) were also called as significant expression QTLs (eQTLs; Bonferroni correction; S3 Table) in GTEx—in keeping with previous studies [14].

### Proteome-by-phenome Mendelian Randomization

Proteome-by-phenome MR was then applied to 54,144 protein-trait pairs obtained from these 64 replicated local-pQTLs and 778 traits obtained from GeneAtlas (UK Biobank) [20], and 68 traits from 20 additional genome-wide association (meta-analysis) studies [24–43] identified through Phenoscanner [21,22] (Fig 1; S4 Table; Methods). Phenoscanner studies were additionally analyzed because, although the UK Biobank cohort is large (~500,000 individuals), for many diseases the number of affected individuals is small, resulting in low statistical power (Methods).

Proteome-by-phenome MR yielded 271 significant protein-trait pairs (FDR <0.05) in GeneAtlas, and 238 significant (FDR <0.05) pairs using Phenoscanner data. Thirty-two of the 64 proteins were causally implicated for one or more traits in GeneAtlas, and 36 of 64 in the Phenoscanner studies’ traits. GeneAtlas and Phenoscanner traits are not mutually exclusive, and some of the Phenoscanner studies included UK Biobank data. Nevertheless, a majority (60%; 38/64) of the proteins were implicated in one or more traits (e.g. IL6R: as discussed below; S5 and S6 Tables).

For some of these inferences, genetic evidence of an association between a protein and phenotype has previously been proposed based simply on physical proximity of the genes to GWA intervals. However, in actually measuring protein products we go well beyond genetic proximity-based annotation of GWA hits: (a) we provide direct evidence that a SNP actually changes the abundance of a protein, and (b) notwithstanding the assumptions of MR, that the change in protein abundance observed is consistent with a causal effect of the protein on outcome trait variation. In addition, notwithstanding the different significance criteria, nearly two-thirds (62%; 318/509) of the significant (FDR <0.05) MR associations between protein and outcome were not matched by significant (p-value <5x10^-8^) association of the DNA variant to outcome.

### Heterogeneity of effect-size estimates

For GeneAtlas results, we use HEIDI to test for heterogeneity of MR effect estimates, and eCAVIAR to assess the colocalization posterior probability (CLPP) of the instrumental variable, within a locus. HEIDI tests for heterogeneity of MR effect between the lead variant (the primary instrument) and those of linked variants. More specifically, it tests the null hypothesis that the observed MR result is consistent with a single causal variant [23], explicitly accounting for the LD structure across the locus. eCAVIAR is a probabilistic method to assess the CLPP, again accounting for LD, that allows for multiple causal variants within a locus.

**Fig 2:**
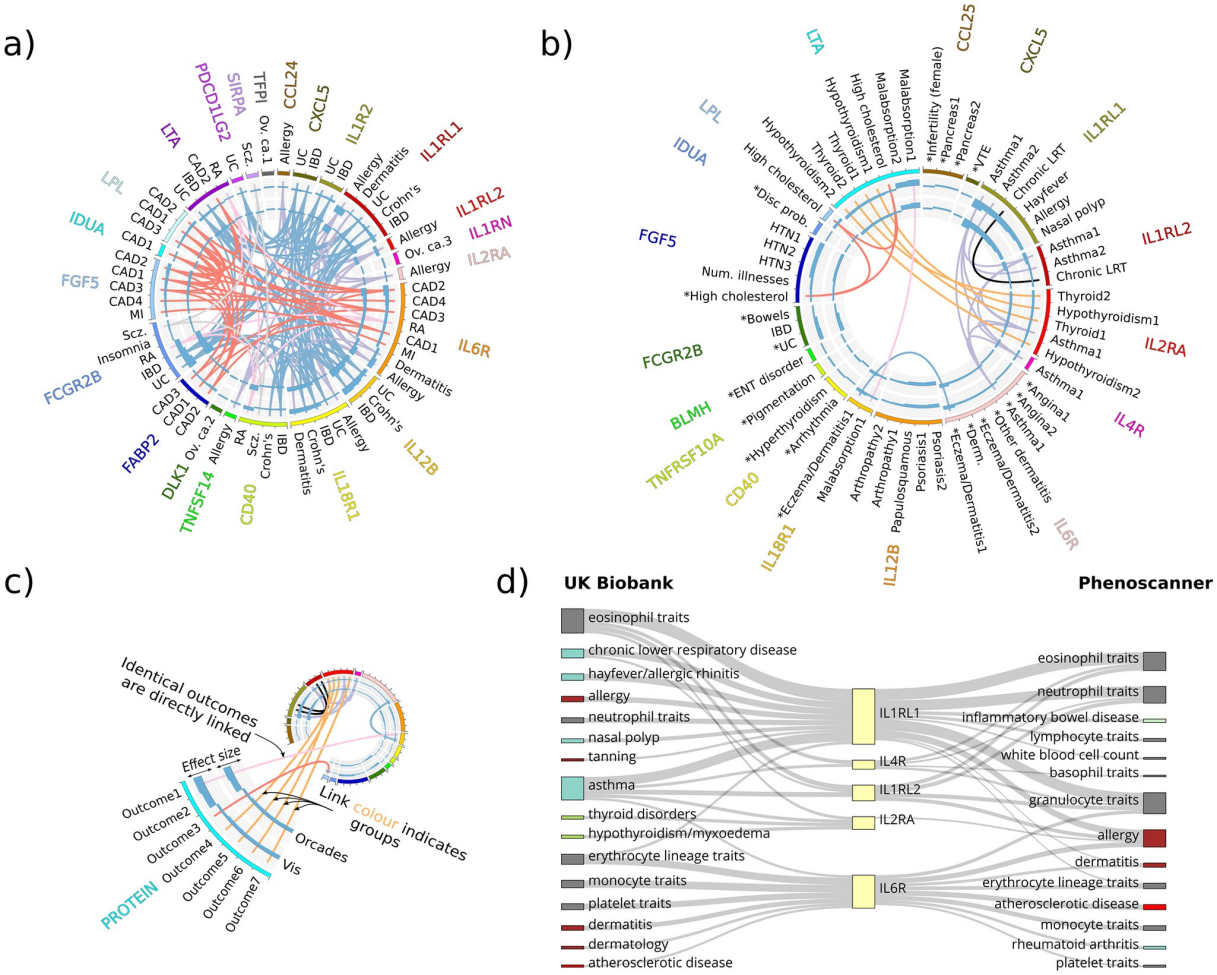
Significant (FDR <0.05) proteome-by-phenome MR protein-outcome causal inferences: Disease subset. MR significant (FDR<5%) protein-disease outcome results. a) All MR significant (FDR<5%) protein-disease outcome results for outcomes from the Phenoscanner [21,22] studies (see key for details). b) All MR significant (FDR<5%) protein-disease outcome results for outcomes from GeneAtlas [20]. An asterisk indicates MR estimates that are *not* significantly heterogeneous upon HEIDI testing (see key for details). c) Key. From the outside in: HGNC symbol of the protein (exposure); disease outcome; key color (matching the protein name in the outer ring); bar chart of the signed squared beta estimate divided by the squared standard error of the MR estimate, using pQTL data from the discovery cohort (CROATIA-Vis); bar chart of the signed squared beta estimate divided by the squared standard error of the MR estimate, using pQTL data from the replication cohort (ORCADES). Central links join identical outcomes for which more than one protein was found to be MR significant. The color of the links indicates similar outcome groups, e.g. thyroid disease. The key to the outcome descriptions is detailed further in S9 and S10 Tables. d) Example concordance (due to sample overlap) plot for all proteins with significant MR evidence in GeneAtlas for causal roles in asthma (IL1RL1, IL1RL2, IL2RA, IL4R, IL6R). GeneAtlas traits are on the left. Phenoscanner traits are on the right. Thickness of connecting lines is proportional to -log_10_(p-value). The Phenoscanner studies included here are derived from [24,26,27,30,38,41–43], of which [26,38,42,43] include at least some part of the UKBB data. However, [26,42,43] use only data from the first phase (~150,000 individuals) genotype release from UK Biobank.

Amongst the GeneAtlas results, 77 of 271 survived the HEIDI heterogeneity testing (p-value >0.05), and 92 of 271 have a CLPP >1% in eCAVIAR (threshold as per the original eCAVIAR paper [44]), with an intersect of 32. These 32 proteins thus have: (1) high-quality evidence of association to a DNA variant that provides congruent predictions for both plasma protein levels and disease risk or trait, and (2) a low risk of pleiotropy, due to the physical proximity of the pQTL to the protein’s gene, survival of the HEIDI test, and a high CLPP in eCAVIAR (S7 Table). These 32 relationships therefore have the most robust evidence that the level of the protein directly alters disease risk or trait. Nevertheless, we emphasize that all 509 causal inferences (271 from GeneAtlas [20] and 238 from studies identified through Phenoscanner [21,22]; Fig 2, and S5 and S6 Tables), even those consistent with heterogeneity (GeneAtlas only), remain potential high-quality drug targets. An appropriate interpretation of this result is that there are 271 potentially causal links identified in GeneAtlas, with additional support for 77 based on results of the HEIDI analysis, 92 based upon eCAVIAR analysis, and 32 with support from both. This may be because the HEIDI heterogeneity test (Fig 1) is susceptible to type I errors (i.e. false positives) in the context of this study. The method can report significant heterogeneity where there is, in fact, none if: (a) there are multiple causal variants present within a locus, or (b) there are differences in the LD structure among the discovery pQTL GWA population (used for lead-SNP selection), the replication pQTL GWA study population (used for effect-size estimation), the outcome trait GWA study population, or that of the LD reference. eCAVIAR may also fail to detect colocalization due to differences in LD structure between the cohorts. In addition, CLPP depends on the complexity of the LD within a locus, complex LD structure can result in low CLPP values: suggesting the possibility of false negative results [44]. Finally, it is worth noting that we applied the HEIDI test in a conservative manner: a significant HEIDI test implies heterogeneity yet we did not apply a multiple testing correction. Applying a Bonferroni correction (271 tests) to the HEIDI p-value, yields 180 of the protein-outcome pairs (rather than 77) as not significantly heterogeneous.

### Tractability of the proteins assessed as therapeutic targets

Of the 32 proteins for which we identified a significant MR association in GeneAtlas (S5 Table), we found 1319 compounds (S8 Table) associated with 10 proteins in ChEMBL. Of these compounds, 10 have already been tested in phase 2, or greater, trials: targeting DLK1, LPL, and LGALS3.

Our results draw causal inference between the plasma concentration of specific proteins and many diseases and outcome phenotypes. For example, we provide supporting evidence for a role of IL4R in asthma, IL2RA in thyroid dysfunction, and IL12B in psoriasis (Fig 2), as well as many cellular phenotypes, such as Transferrin receptor protein 1 (encoded by *TFRC*) in mean corpuscular hemoglobin. Multiple disease endpoints exist to which we have found a MR link and, additionally, for some diseases we have causal links from multiple proteins (Fig 2A and 2B; S5 and S6 Tables).

### Many-to-One: Multiple proteins link to asthma

Asthma is an inflammatory condition affecting the airways. Using GeneAtlas data, our analysis finds 5 proteins—all interleukin receptors—whose levels causally contribute to asthma disease risk: IL1RL1, IL1RL2, IL2RA, IL4R, and IL6R (Fig 2D). Prior links between these proteins and asthma or atopy exist (IL1RL1 [45,46] and IL1RL2 [14], IL2RA [41,47], IL4R [48], and IL6R [41,48–52]), albeit not necessarily strong evidence for a causal link. Of these, IL6R was not significantly heterogeneous in HEIDI testing (p >0.05), and also IL4R if accounting for multiple tests (p >0.05/271). Only IL6R had a CLPP >1% in eCAVIAR. Given the association between eosinophils and asthma, it is worth noting that IL1RL1, IL1RL2, IL2RA, and IL4R are all linked to ‘Eosinophil count’ and ‘Eosinophil percentage’ in GeneAtlas. Whilst not a true replication, due to the use of UK Biobank data in both GeneAtlas and some of the Phenoscanner studies, Fig 2D reveals strong concordance between the MR links identified between the two. Of the 12 Phenoscanner studies reporting significant MR links in this study [24,26–28,30,32,34,37,38,41–43], 5 include UK Biobank data from ~150,000 individuals [26,32,34,42,43], and one uses the full UK Biobank release [38].

### One-to-Many: Linking IL6R levels to atopy, rheumatoid arthritis, and coronary artery disease

We also found evidence for a causal association between plasma IL6R abundance and coronary artery disease (CAD), atopy, and rheumatoid arthritis (Fig 2, S5 and S6 Tables). We note previous support for these inferences: for example, tocilizumab (a humanized monoclonal antibody against IL6R protein) is in clinical use for treating rheumatoid arthritis [53], prior MR evidence has linked elevated levels of soluble IL6R to reduced cardiovascular disease [54,55], and, as discussed above, there is previous genetic evidence of a link between IL6R and atopy [41,48–52].

### SHPS1 and schizophrenia

**Fig 3:**
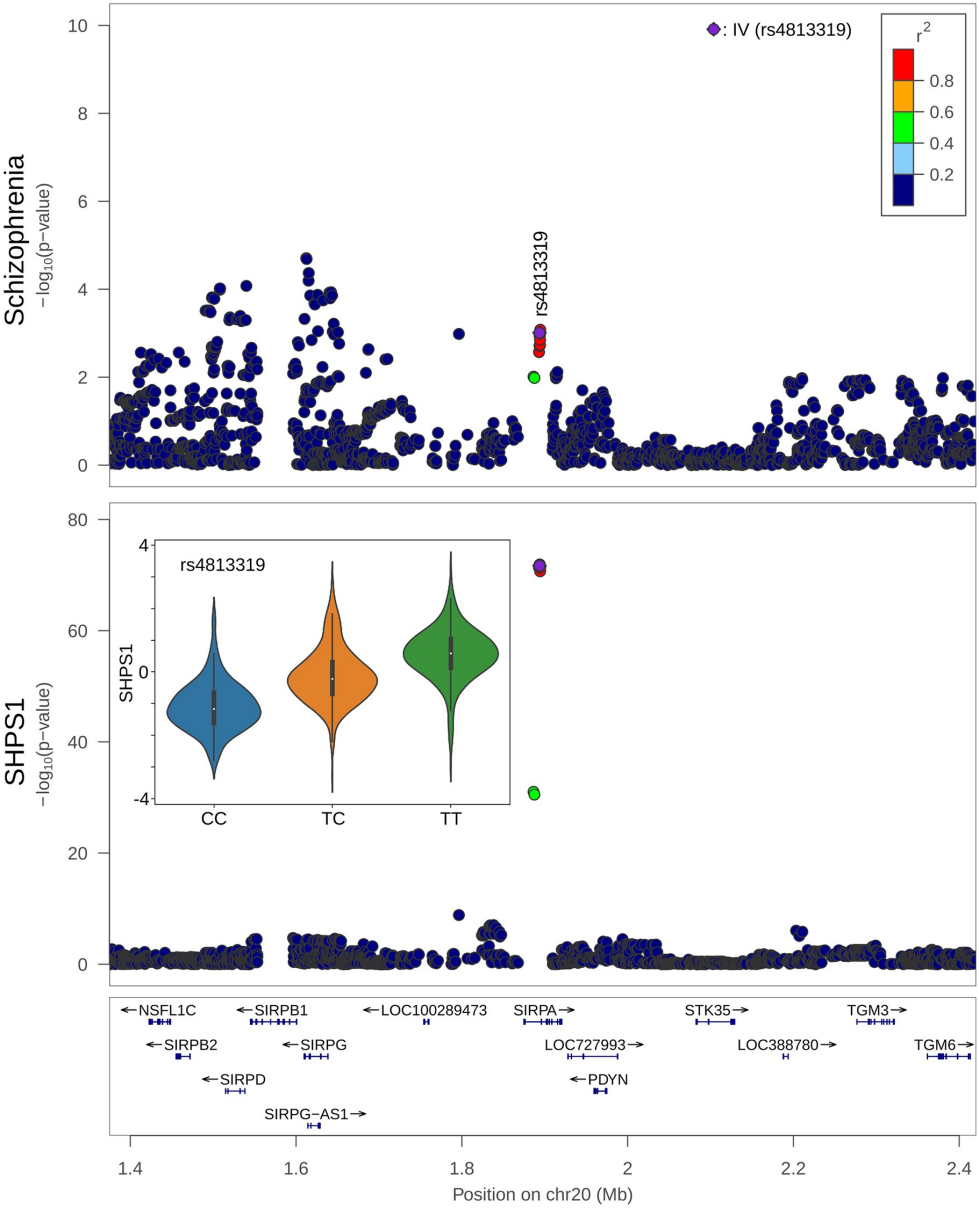
Co-localization of SHPS1 (encoded by *SHPS1*: Synonym *SIRPA*) and schizophrenia DNA associations. Upper panel, LocusZoom [56] of the region surrounding *SHPS1* and the associations with schizophrenia [28]; lower panel, associations with SHPS1. Lower panel inset, the relative concentration of SHPS1 across the 3 genotypes of rs4813319 –the DNA variant used as the instrumental variable (IV) in the MR analysis: CC, CT, and TT.

Three proteins were implicated in the pathogenesis of schizophrenia: (i) Tyrosine-protein phosphatase non-receptor type substrate 1 (SHPS1; *SIRPA*)–Fig 3, (ii) Tumor necrosis factor receptor superfamily member 5 (*CD40*), and (iii) Low affinity immunoglobulin gamma Fc region receptor II-b (*FCGR2B*).

Focusing on SHPS1, it is highly expressed in the brain, especially in the neuropil (a dense network of axons, dendrites, and microglial cell processes) in the cerebral cortex (https://v18.proteinatlas.org/ENSG00000198053-SIRPA/tissue [57–59]; accessed 01 Apr 2019), and co-localizes with CD47 at dendrite-axon contacts [60]. Mouse models in which the *SHPS1* gene is disrupted exhibit many nervous system abnormalities, such as reduced long term potentiation, abnormal synapse morphology and abnormal excitatory postsynaptic potential (MGI: 5558020 [61]; http://www.informatics.jax.org/; v6.13; accessed 01 Apr 2019). Other mouse and rat models link CD47 to sensorimotor gating and social behavior phenotypes [62–66]. In addition, SHPS1 mediates activity-dependent synapse maturation [61] and may also have a role as a “don’t eat me” signal to microglia [67]. SHPS1 levels tend to be lower in the dorsolateral prefrontal cortex of schizophrenia patients [68]. Finally, the observed effect of SHSP1 on schizophrenia was not significantly heterogeneous in the results of the Schizophrenia Working Group of the Psychiatric Genomics Consortium (2014) (p-value 0.53).

### FABP2 and coronary artery disease

Four other proteins, in addition to IL6R, were identified as contributing to CAD pathogenesis, namely FABP2, FGF5, LPL, and LTA (Fig 2). FGF5, LPL, IL6R, and LTA had been implicated previously [26,69,70], whereas FABP2 had more limited prior evidence for its involvement.

pQTL analysis identified two lead DNA variants in close proximity (<150kb) to the *FABP2* gene. Using SNP rs17009129, we find a causal link between FABP2 abundance and CAD (p-value 1.1x10^−4^; FDR <0.05; β_MR_ -0.11; se_MR_ 0.028; β_MR_ and se_MR_ units: log(OR)/standard deviation of residualised protein concentration) without significant heterogeneity (p-value 0.24) which suggests shared causal genetic control. Furthermore, a second independent SNP (LD r^2^ <0.2; rs6857105) replicates this observation (MR p-value 5.0x10^−4^; HEIDI p-value 0.34; β_MR_ -0.17; se_MR_ 0.047). Both SNPs (rs17009129, and rs6857105) fell below genome-wide significance (p-value <5x10^−8^) in the full meta-analysis of van der Harst [38] on CAD. Consequently, this is the first time, to our knowledge, that variants associate with *FABP2* abundance have been demonstrated to contribute causally to CAD pathogenesis.

## Discussion

Proteome-by-phenome MR efficiently and robustly yields evidence for proteins as drug targets. It offers a data-driven approach to drug discovery using population-level data, and quantifies the strength of evidence for causation. Previous studies have made successful forays into the use of pQTL in mapping protein variation onto disease [12–18], and both the coverage of the proteome and the availability of disease and trait GWA study results are ever increasing. By using the lead variants of locally-acting pQTLs as instrumental variables, we focused specifically on a subset of functionally relevant variants for those proteins under study: this choice reduced the multiple testing burden when compared to genome-wide scans for associations of the outcome trait.

A potential problem with antibody- and aptamer-based assays is that any perturbation to binding, such as a change to an epitope, appears incorrectly as a change in abundance. In the absence of a well-defined reference, we cannot exclude the possibility that some of the pQTL we have called indicate epitope changes rather than changes in protein abundance. However, in each case, a bona fide biological association does exist between the genetic variant and the protein. With respect to MR, this would change the biological interpretation of the exposure only: protein abundance or sequence isoform, for example.

In addition, proteome-by-phenome MR has inherent limitations. First, a true positive MR association in our analysis implies that any intervention to replicate the effect of a given genotype would alter the relevant phenotype. Nevertheless, this association is informative neither of the time interval, during development for example, nor the anatomical location in which an intervention would need to be delivered. Second, pleiotropic effects cannot be excluded entirely without (unachievable) quantification of every mediator. Third, the abundance of a protein in plasma may be an imperfect proxy for the effect of a drug targeting that protein at the level of a whole organism. Finally, plasma abundance does not necessarily reflect activity. For example, a variant may cause expression of high levels of an inactive form of a protein. Or, for proteins with both membrane-bound and unbound forms, the MR direction of effect observed from quantifying soluble protein abundance may not reflect that of membrane-bound protein. For many membrane-bound proteins, a soluble (often antagonistic) form exists that is commonly produced through alternative splicing or proteolytic cleavage of the membrane-bound form. Based on 1,000 Genomes [71,72] data, the variant we use to predict IL6R level, rs61812598, for example, is in complete LD with the missense variant rs2228145 whose effects on proteolytic cleavage of the membrane-bound form and alternative splicing have been examined in detail [73]. Carriers of the 358Ala allele at rs2228145 tend to have increased soluble IL6R but reduced membrane-bound IL6R in a number of immune cell types. Differences between the effects of soluble and membrane-bound forms of a protein may be widespread. For example, dupilumab is a monoclonal antibody that targets IL4R, a key component of both IL4 and IL13 signaling. It is currently under investigation for the treatment of asthma and has shown promising results in both eosinophilic and non-eosinophilic asthma [74,75]. Based on our results, we would have predicted that increased levels of IL4R result in a lower risk of asthma (S5 Table). This is in contrast to the direction-of-effect due to dupilumab administration. However, as with IL6R, IL4R has both a soluble and a membrane-bound form. Encouragingly, despite this, a relationship between dupilumab and asthma remains plausible—as evidenced by the 14 recently completed or ongoing clinical trials to assess the efficacy and safety of dupilumab in asthma (as of 26 March 2019, ClinicalTrials.gov).

As well as its utility in identifying potential therapeutic targets for drug development, proteome-by-phenome MR also allows for an assessment of potential off-target effects of existing pharmacological targets. For example, we predict an effect of IL4R modulation on eosinophil count and percentage. This is an association already realized in one of the phase II clinical trials investigating dupilumab in asthma: a rise in eosinophil count was observed for some patients, even leading to the withdrawal of one patient from the study [74].

In summary, we have identified dozens of plausible causal links by conducting GWA of 249 proteins, followed by phenome-wide MR using replicated locally-acting pQTLs of 64 proteins. The approach is statistically robust, relatively inexpensive, and high-throughput. 54,144 protein-outcome links were assessed and 509 significant (FDR <0.05) links identified: including anthropometric measures, hematological parameters, and diseases. Opportunities to discover larger sets of plausible causal links will increase as study sizes and pQTL numbers grow. Indeed, whole-proteome versus Biobank GWA Atlas studies will likely become feasible as pQTL measurement technologies mature further.

## Methods

### Ethics statement

ORCADES: The study was approved by Research Ethics Committees in Orkney and Aberdeen (North of Scotland REC, 26/11/2003).

CROATIA-Vis: The study received approval from the relevant ethics committees in Scotland (South East Scotland Research Ethics Committee, REC reference: 11/AL/0222) and Croatia (University of Split School of Medicine Ethics committee, Class:003-08/11-03/-005 No.: 2181-198-03-04/10-11-0008).

All participants gave written informed consent and both studies complied with the tenets of the Declaration of Helsinki.

#### Cohort description

From the islands of Orkney (Scotland) and Vis (Croatia) respectively, the ORCADES [76] and CROATIA-Vis [77,78] studies are of two isolated population cohorts that are both genotyped and richly phenotyped.

The Orkney Complex Disease Study (ORCADES) is a family-based, cross-sectional study that seeks to identify genetic factors influencing cardiovascular and other disease risk in the isolated archipelago of the Orkney Isles in northern Scotland [76]. Genetic diversity in this population is decreased compared to Mainland Scotland, consistent with the high levels of endogamy historically. 2,078 participants aged 16–100 years were recruited between 2005 and 2011, most having three or four grandparents from Orkney, the remainder with two Orcadian grandparents. Fasting blood samples were collected and many health-related phenotypes and environmental exposures were measured in each individual.

The CROATIA-Vis study includes 1,008 Croatians, aged 18–93 years, who were recruited from the villages of Vis and Komiza on the Dalmatian island of Vis during spring of 2003 and 2004. They underwent a medical examination and interview, led by research teams from the Institute for Anthropological Research and the Andrija Stampar School of Public Health, (Zagreb, Croatia). All subjects visited the clinical research center in the region, where they were examined in person and where fasting blood was drawn and stored for future analyses. Many biochemical and physiological measurements were performed, and questionnaires of medical history as well as lifestyle and environmental exposures were collected.

#### Genotyping

Chromosomes and positions reported in this paper are from GRCh37 throughout. Genotyping of the ORCADES cohort was performed on the Illumina Human Hap 300v2, Illumina Omni Express, and Illumina Omni 1 arrays; that of the CROATIA-Vis cohort used the Illumina HumanHap300v1 array.

The genotyping array data were subject to the following quality control thresholds: genotype call-rate 0.98, per-individual call-rate 0.97, failed Hardy-Weinberg test at p-value <1x10^−6^, and minor allele frequency 0.01; genomic relationship matrix and principal components were calculated using GenABEL (1.8–0) [79] and PLINK v1.90 [80,81].

Assessment for ancestry outliers was performed by anchored PCA analysis when compared to all non-European populations from the 1,000 Genomes project [71,72]. Individuals with a mean-squared distance of >10% in the first two principal components were removed. Genotypes were phased using Shapeit v2.r873 and duoHMM [82] and imputed to the HRC.r1-1 reference panel [83]. 278,618 markers (Hap300) and 599,638 markers (Omni) were used for the imputation in ORCADES, and 272,930 markers for CROATIA-Vis.

#### Proteomics

Plasma abundance of 249 proteins was measured in two European cohorts using Olink Proseek Multiplex CVD2, CVD3, and INF panels. All proteomics measurements were obtained from fasting EDTA plasma samples. Following quality control, there were 971 individuals in ORCADES, and 887 individuals in CROATIA-Vis, who had genotype and proteomic data from Olink CVD2, 993 and 899 from Olink CVD3, and 982 and 894 from Olink INF. The Olink Proseek Multiplex method uses a matched pair of antibodies for each protein, linked to paired oligonucleotides. Binding of the antibodies to the protein brings the oligonucleotides into close proximity and permits hybridization. Following binding and extension, these oligonucleotides form the basis of a quantitative PCR reaction that allows relative quantification of the initial protein concentration [84]. Olink panels include internal and external controls on each plate: two controls of the immunoassay (two non-human proteins), one control of oligonucleotide extension (an antibody linked to two matched oligonucleotides for immediate proximity, independent of antigen binding) and one control of hybridized oligonucleotide detection (a pre-made synthetic double stranded template), as well as an external, between-plate, control (http://www.olink.com/; accessed: 19th June 2016).

Prior to analysis, we excluded proteins with fewer than 200 samples with measurements above the limit of detection of the assay. Of the 268 unique proteins reported by Olink, 253 passed this threshold in ORCADES, and 252 in CROATIA-Vis, with an intersect of 251 proteins. Protein values were inverse-normal rank-transformed prior to subsequent analysis.

The subunits of IL27 are not distinguished in Olink’s annotation (Q14213, *EBI3*; and Q8NEV9, *IL27*). However, it has only one significant locus, local to the *EBI3* gene (lead variant, rs60160662, is within 16kb). Therefore, *EBI3* (Q14213) was selected as representative for this protein when discussing pQTL location (local/distal) so as to avoid double counting.

The CVD2, CVD3, and INF panels are commercially available from Olink. The proteins on these panels were selected by Olink due to *a priori* evidence of involvement in cardiovascular and inflammatory processes. Two proteins, CCL20 and BDNF, have been removed at the request of Olink (due to issues with the assay).

#### Detection of pQTL

Genome-wide association of these proteins was performed using autosomes only. Analyses were performed in three-stages. (1) a linear regression model was used to account for participant age, sex, genotyping array (ORCADES only), proteomics plate, proteomics plate row, proteomics plate column, length of sample storage, season of venepuncture (ORCADES only), and the first 10 principal components of the genomic relationship matrix. Genotyping array and season of venepuncture are invariant in CROATIA-Vis and therefore were not included in the model. (2) Residuals from this model were corrected for relatedness, using GenABEL’s [79] polygenic function and the genomic relationship matrix, to produce GRAMMAR+ residuals. Outlying GRAMMAR+ residuals (absolute z-score >4) were removed and the remainder rank-based inverse-normal transformed. (3) Genome-wide association testing was performed using REGSCAN v0.5 [85].

Genome-wide association results were clumped by linkage disequilibrium using PLINK v1.90 [80,81]. Biallelic variants within ±5Mb and *r*^2^ >0.2 to the lead variant (smallest p-value at the locus) were clumped together, and the lead variant is presented. *r*^2^ was derived from all European populations in 1,000 Genomes [71,72].

We have chosen to describe pQTL as *local-* or *distant-* so as to distinguish naming based on genomic location from that based on mode of action i.e. *cis-* (acting on the same DNA molecule) and *trans-* (acting via some diffusible mediator). That is, most *local-* variation may well act in *cis* but not necessarily so.

#### Mendelian Randomization

In the context of proteome-by-phenome MR, a DNA variant (a single nucleotide polymorphism in this case) that influences plasma protein level is described as an ‘instrumental variable’, the protein as the ‘exposure variable’, and the outcome phenotype as the ‘outcome variable’.

The lead-SNP with the lowest p-value meeting the following criteria was used as the instrumental variable for each protein:

1. Minor allele frequency >1% in both ORCADES and CROATIA-Vis cohorts.
2. An imputation info score (SNPTEST v2) of >0.95 in both ORCADES and CROATIA-Vis.
3. Located within ±150kb of the gene coding for the protein (start and end coordinates of the gene as defined by Ensembl GRCh37 [86]).
4. Significant (as defined below) SNP:protein link in both the discovery and replication cohorts.

Lead-SNP selection was performed using the discovery (CROATIA-Vis; p-value <5x10^-8^) cohort; replication was defined based on a Bonferroni correction for the number of significant lead-SNPs present in the discovery cohort (CROATIA-Vis). In order to avoid a ‘winner’s curse’, genome-wide association effect size estimates and standard errors from the replication cohort (ORCADES) were used for MR.

We perform MR as a ratio of expectations, using up to second-order partial derivatives of the Taylor series expansion for effect size estimates, and up to first-order for standard errors (Delta method) [87]:

$$\beta_{YX}\approx \frac{\beta_{YZ}}{\beta_{XZ}}\left(1+\frac{se_{XZ}^{2}}{\beta_{XZ}^{2}}\right)$$ (1)

$$se_{YX}\approx \sqrt{\frac{se_{YZ}^{2}}{\beta_{XZ}^{2}}+\frac{\beta_{YZ}^{2}\times se_{XZ}^{2}}{\beta_{XZ}^{4}}}$$ (2)

$$p_{YX}\approx 2\Phi \left(\frac{-\left|\beta_{YX}\right|}{se_{YX}}\right)$$ (3)

where *β*_*ij*_ is the causal effect of *j* on *i*, *se*_*ij*_ is the standard error of the causal effect estimate of *j* on *i*; subscript *X* is the exposure, *Y* the outcome trait, and *Z* the instrumental variable. Φ is the cumulative density function of the standard normal distribution. This method is identical to that of SMR [23] apart from the second term in the bracket of Eq 1 (resulting from the inclusion of second-order partial derivatives). An FDR of <0.05 was considered to be significant. FDR estimations were performed separately on those results derived from GeneAtlas and those derived from studies in Phenoscanner.

#### DNA variant to trait association: GeneAtlas

UK Biobank has captured a wealth of information on a large—approximately 500,000 individuals—population cohort that includes anthropometry, hematological traits, and disease outcomes. All 778 outcome traits from UK Biobank in GeneAtlas (http://geneatlas.roslin.ed.ac.uk/; Canela-Xandri et al. (2018) [88]) were included. The analysis method of all 778 traits was as described for 717 in Canela-Xandri et al. (2017) [20]. For each protein, the lead (lowest DNA variant-protein association p-value in the discovery cohort) biallelic (Phase 3, 1,000 Genomes [71,72]) variant meeting the criteria above and an imputation info score >0.95 in UK Biobank, was selected for each protein, and MR performed.

#### DNA variant to trait association: Phenoscanner

Phenoscanner [21,22] was used to highlight existing GWA studies for inclusion. For each protein, the lead (lowest DNA variant-protein association p-value in the discovery cohort) biallelic (1,000 Genomes [71,72]) meeting the criteria above was selected. rs545634 was not found in the Phenoscanner database and was therefore replaced with the second most significant variant meeting the above criteria: chr1:15849003. Phenoscanner was run with the following options: Catalogue: ‘Diseases & Traits’, p-value cut-off: ‘1’, Proxies: ‘None’, Build ‘37’. The results from those studies that returned a value for all input variants were kept and MR performed. Phenoscanner (http://www.phenoscanner.medschl.cam.ac.uk/information/; accessed 25 Sep 2018) state that they report all SNPs on the positive strand. Given this, alleles were harmonized as required. No attempt to harmonize based on allele frequency was made; therefore, the direction of effect of C/G and A/T SNPs should be interpreted with care. Results from 20 additional studies were obtained, corresponding to 68 outcomes.

#### HEIDI

Heterogeneity in dependent instruments (HEIDI) analysis [23], is a method of testing whether the MR estimates obtained using variants in linkage disequilibrium with the lead variant are consistent with a single causal variant at a given locus (Fig 1D). HEIDI analysis was performed using software provided at https://cnsgenomics.com/software/smr/ (accessed 28 Aug 2018; v0.710). We used pQTL data from ORCADES for assessment as the exposure. Biallelic variants from the 1,000 Genomes [71,72] (European populations: CEU, FIN, GBR, IBS, and TSI) were used as the linkage disequilibrium reference. We used the default ‘cis-window’ of 2000kb, and a maximum number of variants of 20 (as is the default value for the software).

We performed HEIDI analysis of all exposure-outcome links that were found to be significant (FDR <0.05) using outcomes from GeneAtlas (n = 271), as well as links found to be MR significant (FDR <0.05) with CAD from the meta-analysis of van der Harst [38], and for SHPS1 and schizophrenia [28].

We applied the following filters for variants to be included in the analysis: minor allele frequency MAF >0.01 and, in the GeneAtlas and ORCADES data, an imputation info score of >0.95.

#### eCAVIAR

eCAVIAR [44] is a method for assessing the colocalization posterior probability (CLPP) for two traits at a locus, whilst allowing for multiple causal variants. We ran eCAVIAR with a maximum of 5 causal variants per locus and defined a locus as per the original eCAVIAR paper [44]: 50 SNPs up- and down-stream of the relevant variable (the instrumental variable in this case). eCAVIAR was run using software provided at https://github.com/fhormoz/caviar/ (accessed 12 Mar 2020; v2.2). As with HEIDI, we used pQTL data from ORCADES for assessment as the exposure, biallelic variants from the 1,000 Genomes [71,72] as an LD reference, and applied identical filters for variant inclusion.

We performed eCAVIAR analysis of all exposure-outcome links that were found to be significant (FDR <0.05) using outcomes from GeneAtlas (n = 271).

### Comparison to eQTL

Result for all SNP:gene pairs analyzed in whole blood were downloaded from GTEx [19] (v7) from the GTEx Portal (https://gtexportal.org/; accessed 04 Sep 2019). Results were extracted for the instrumental variables and the genes encoding their proteins for the 64 proteins for which an instrumental variable was successfully identified in this study. Matching was based on Ensembl Gene ID, and variant chromosome, position, and alleles (GRCh37).

### Comparison to plasma pQTL using an orthogonal, aptamer-based, method

The supplementary data files for Sun et al. [14] were downloaded on 04 Sep 2019. From Supplementary Table 4, pQTL identified were extracted for the 64 proteins for which an instrumental variable was successfully identified in this study. Proteins were matched based on an exact UniProtID match. The LD (r^2^) between the lead locally-acting (as defined above) and ‘cis-acting’ (as defined by Sun et al.) SNP identified for each protein was calculated using the European populations from the 1,000 Genomes project (as described above) using PLINK v1.90 [80,81].

### Links to existing drug therapies

Protein names were matched to ChEMBL IDs using the UniProtID mapping API (https://www.uniprot.org/help/api_idmapping; accessed 27 Oct 2019). ChEMBL [89] was searched programmatically using the ChEMBL web resource client in Python 3.6 (https://github.com/chembl/chembl_webresource_client; accessed 27 Oct 2019).

## Supporting information

S1 Table List of pQTLs (linkage disequilibrium clumped). List of lead SNPs for each protein following linkage disequilibrium (LD) clumping, together with replication information. Biallelic variants within ±5Mb and r^2^ >0.2 to the lead variant (smallest p-value at the locus) were clumped together. European populations in 1,000 Genomes [71,72] were used as the LD reference. Columns are: ‘hgnc_symbol’: HUGO gene naming consortium symbol of the exposure (protein); ‘snpid’: ‘chr’_‘pos’; ‘rsid’: rsID; ‘chr’: chromosome (GRCh37) of the SNP; ‘pos’: position (GRCh37) of the SNP; ‘a1’: effect allele; ‘a0’: other allele; ‘n_pri’: number of individuals in the primary cohort (CROATIA-Vis); ‘freq1_pri’: frequency of the effect allele in the primary cohort (CROATIA-Vis); ‘beta1_pri’: beta estimate of the effect allele in the primary cohort (CROATIA-Vis); ‘se_pri’: standard error of ‘beta1_pri‘ in the primary cohort (CROATIA-Vis); ‘p_pri’: p-value of ‘beta1_pri‘ and ‘se_pri’; ‘info_pri’: SNPTEST (v2) info of the imputation in the primary cohort (CROATIA-Vis); ‘r2_pri’: coefficient of determination of the regression in the primary cohort (CROATIA-Vis); ‘n_sec’: as for the primary cohort (CROATIA-Vis) but in the secondary cohort (ORCADES); ‘freq1_sec’: as for the primary cohort (CROATIA-Vis) but in the secondary cohort (ORCADES); ‘beta1_sec’: as for the primary cohort (CROATIA-Vis) but in the secondary cohort (ORCADES); ‘se_sec’: as for the primary cohort (CROATIA-Vis) but in the secondary cohort (ORCADES); ‘p_sec’: as for the primary cohort (CROATIA-Vis) but in the secondary cohort (ORCADES); ‘info_sec’: as for the primary cohort (CROATIA-Vis) but in the secondary cohort (ORCADES); ‘r2_sec’: as for the primary cohort (CROATIA-Vis) but in the secondary cohort (ORCADES); ‘uniprot_swissprot’: UniProtID of the exposure (protein), see http://www.uniprot.org/; ‘ensembl_gene_id’: Ensembl gene ID (GRCh37; see http://grch37.ensembl.org/index.html) of the gene-of-origin of the protein; ‘chromosome_name’: chromosome (GRCh37) of the gene of the protein, as per Ensembl GRCh37; ‘start_position’: start position (GRCh37) of the gene of the protein, as per Ensembl GRCh37; ‘end_position’: end position (GRCh37) of the gene of the protein, as per Ensembl GRCh37; ‘description’: HUGO gene naming consortium description of the exposure (protein); ‘replicated_pqtl’: is the lead SNP of the cluster (as identified in the primary cohort) replicated in the secondary cohort (Bonferroni correction for multiple testing. TRUE if it is; FALSE if not); ‘within_gene_plus_flank_tol’: is the SNP within the gene-of-origin of the protein +/- 150kb (TRUE is it is; FALSE if not).
(TSV)

S2 Table Comparison of the lead-SNPs identified here and those identified using an orthogonal, aptamer-based assay. Aptamer-based assay results are those of Sun et al. [14]. Columns are ‘hgnc_symbol’: the HGNC symbol corresponding to the UniProtID; ‘exposure’: the UniProtID of the protein; ‘rsid_olink’: the rsID of the lead-SNP from this study; ‘chr_olink’: the chromosome, GRCh37, of the lead-SNP from this study; ‘pos_olink’: the position, GRCh37, of the lead-SNP from this study; ‘a1_olink’: allele 1 of the lead-SNP from this study; ‘a0_olink’: allele 0 of the lead-SNP from this study; ‘rsid_sun’: the rsID of the lead-SNP from Sun et al.; ‘chr_sun’: the chromosome, GRCh37, of the lead-SNP from Sun et al.; ‘pos_sun’: the position, GRCh37, of the lead-SNP from Sun et al.; ‘a1_sun’: allele 1 of the lead-SNP from Sun et al.; ‘a0_sun’: allele 0 of the lead-SNP from Sun et al.; ‘ld_r2’: the linkage disequilibrium (r^2^) of the two SNPs, as measured in the European individuals from 1,000 Genomes (Methods).
(TSV)

S3 Table Comparison of the lead-SNPs identified here and eQTL. eQTL data derived from ‘Whole blood’ from GTEx [19] (v7). Bonferroni correction 0.05/54. Columns are ‘hgnc_symbol’: the HGNC symbol corresponding to the UniProtID; ‘rsid’: rsID of the SNP; ‘chr’: chromosome of the SNP, GRCh37; ‘pos’: position of the SNP, GRCh37; ‘a1’: the effect allele; ‘a0’: the other allele; ‘uniprot’: UniProtID of the protein; ‘n_protein_pri’: number of individuals in the primary protein cohort (CROATIA-Vis); ‘freq1_protein_pri’: frequency of the effect allele in the primary protein cohort (CROATIA-Vis); ‘beta1_protein_pri’: effect-size estimate in the primary protein cohort (CROATIA-Vis); ‘se_protein_pri’: standard error of ‘beta1_protein_pri’; ‘p_protein_pri’: p-value of ‘beta1_protein_pri’ and ‘se_protein_pri’; ‘info_protein_pri’: SNPTEST (v2) imputation info score in the primary protein cohort (CROATIA-Vis); ‘n_protein_sec’: as for the primary cohort (CROATIA-Vis) but in the secondary cohort (ORCADES); ‘freq1_protein_sec’: as for the primary cohort (CROATIA-Vis) but in the secondary cohort (ORCADES); ‘beta1_protein_sec’: as for the primary cohort (CROATIA-Vis) but in the secondary cohort (ORCADES); ‘se_protein_sec’: as for the primary cohort (CROATIA-Vis) but in the secondary cohort (ORCADES); ‘p_protein_sec’: as for the primary cohort (CROATIA-Vis) but in the secondary cohort (ORCADES); ‘info_protein_sec’: as for the primary cohort (CROATIA-Vis) but in the secondary cohort (ORCADES); ‘ensembl_gene_id’: Ensembl gene ID corresponding to the protein; ‘pval_nominal_gtex’: nominal p-value in GTEx (v7) whole blood; ‘slope_gtex’: effect-size estimate in GTEx (v7) whole blood; ‘slope_se_gtex’: standard error of ‘slope_gtex’ in GTEx (v7) whole blood; ‘pval_nominal_threshold_gtex’: nominal p-value threshold for calling a variant-gene pair significant for the gene in GTEx (v7) whole blood; ‘min_pval_nominal_gtex’: smallest nominal p-value for the gene in GTEx (v7) whole blood; ‘pval_beta’: beta-approximated permutation p-value for the gene in GTEx (v7) whole blood.
(TSV)

S4 Table Additional studies identified using Phenoscanner. Table of the additional studies (and outcome traits) identified through Phenoscanner [21,22]. Note that ‘Coronary artery disease’ was included from van der Harst et al. [38] both with and without the inclusion of data from UK Biobank. Columns are ‘Outcome’: trait under study; ‘PMID’: PubMed ID of the study; ‘First author’: First author the publication; ‘Year’: year of publication of the study; ‘Paper title’: title of the study.
(TSV)

S5 Table Mendelian Randomization results from GeneAtlas. Table of the all significant (FDR <0.05) Mendelian Randomization (MR) results using data from GeneAtlas [20]. pQTL for both cohorts are included, however, in order to avoid a ‘winner’s curse’, MR was conducted using data from the secondary protein cohort (ORCADES). Columns are ‘hgnc_symbol’: HUGO Gene Nomenclature Committee symbol of the exposure protein; ‘outcome_description’: description of the UK biobank outcome from GeneAtlas; ‘rsid’: rsID; ‘snpid’: ‘chr’_‘pos’; ‘chr’: chromosome (GRCh37); ‘pos’: position (GRCh37); ‘a1’: effect allele; ‘a0’: other allele; ‘exposure’: UniProtID of the protein; ‘ensembl_gene_id’: Ensembl (GRCh37) gene ID of the exposure protein; ‘n_exposure_pri’: number of individuals in the primary protein cohort (CROATIA-Vis); ‘freq1_exposure_pri’: frequency of the effect allele in the primary protein cohort (CROATIA-Vis); ‘beta1_exposure_pri’: regression coefficient (per additional effect allele) in the primary protein cohort (CROATIA-Vis); ‘se_exposure_pri’: standard error of ‘beta1_exposure_pri’; ‘p_exposure_pri’: p-value of ‘beta1_exposure_pri’ and ‘se_exposure_pri’; ‘info_exposure_pri’: SNPTEST (v2) imputation info score in the primary protein cohort (CROATIA-Vis); ‘n_exposure_sec’: as for the primary cohort (CROATIA-Vis) but in the secondary cohort (ORCADES); ‘freq1_exposure_sec’: as for the primary cohort (CROATIA-Vis) but in the secondary cohort (ORCADES); ‘beta1_exposure_sec’: as for the primary cohort (CROATIA-Vis) but in the secondary cohort (ORCADES); ‘se_exposure_sec’: as for the primary cohort (CROATIA-Vis) but in the secondary cohort (ORCADES); ‘p_exposure_sec’: as for the primary cohort (CROATIA-Vis) but in the secondary cohort (ORCADES); ‘info_exposure_sec’: as for the primary cohort (CROATIA-Vis) but in the secondary cohort (ORCADES); ‘outcome’: outcome code of the UK biobank outcome from GeneAtlas; ‘beta1_outcome’: beta of the effect allele on the outcome in GeneAtlas; ‘se_outcome’: standard error of ‘beta1_outcome’; ‘p_outcome’: p-value corresponding to ‘beta1_outcome’ and ‘se_outcome’; ‘info_outcome’: imputation info score in UK Biobank; ‘freq1_outcome’: frequency of the effect allele in UK Biobank; ‘beta_mr_delta_sec’: beta value using the delta MR method (using up to second order partial derivatives; See the appendix of Lynch and Walsh for further information) using estimates from the secondary cohort; ‘se_mr_delta_sec’: standard error of ‘beta_mr_delta_sec’ using the delta MR method (using up to first order partial derivatives; See the appendix of Lynch and Walsh for further information) using estimates from the secondary cohort; ‘p_mr_delta_sec’: p-value corresponding to ‘beta_mr_delta_sec’ and ‘se_mr_delta_sec’; ‘fdr_sig_mr_delta_sec’: significance of ‘p_mr_delta_sec’ at a False Discovery Rate (FDR) of <5%. True / False.
(TSV)

S6 Table Mendelian Randomization results from studies identified using Phenoscanner. Table of all Mendelian Randomization results using data acquired through Phenoscanner [21,22]. pQTL for both cohorts are included, however, in order to avoid a ‘winner’s curse’, MR was conducted using data from the secondary protein cohort. Columns are ‘hgnc_symbol’: HUGO Gene Nomenclature Committee symbol of the exposure protein; ‘trait’: outcome trait description; ‘snp’: chr‘chr’:‘pos’; ‘rsid’: rsID; ‘chr’: chromosome (GRCh37); ‘pos’: position (GRCh37); ‘a1’: effect allele; ‘a0’: other allele; ‘exposure’: UniProtID of the protein; ‘n_exposure_pri’: number of individuals in the primary protein cohort (CROATIA-Vis); ‘freq1_exposure_pri’: frequency of the effect allele in the primary protein cohort (CROATIA-Vis); ‘beta1_exposure_pri’: regression coefficient (per additional effect allele) in the primary protein cohort (CROATIA-Vis); ‘se_exposure_pri’: standard error of ‘beta1_exposure_pri’; ‘p_exposure_pri’: p-value of ‘beta1_exposure_pri’ and ‘se_exposure_pri’; ‘info_exposure_pri’: SNPTEST (v2) imputation info score in the primary protein cohort; ‘n_exposure_sec’: as for the primary cohort (CROATIA-Vis) but in the secondary cohort (ORCADES); ‘freq1_exposure_sec’: as for the primary cohort (CROATIA-Vis) but in the secondary cohort (ORCADES); ‘beta1_exposure_sec’: as for the primary cohort (CROATIA-Vis) but in the secondary cohort (ORCADES); ‘se_exposure_sec’: as for the primary cohort (CROATIA-Vis) but in the secondary cohort (ORCADES); ‘p_exposure_sec’: as for the primary cohort (CROATIA-Vis) but in the secondary cohort (ORCADES); ‘info_exposure_sec’: as for the primary cohort (CROATIA-Vis) but in the secondary cohort (ORCADES); ‘ensembl_gene_id’: Ensembl (GRCh37) gene ID of the exposure protein; ‘study’: name of the consortium/lead author of the outcome study; ‘pmid’: PubMed ID of the outcome study; ‘ancestry’: ancestry of the population within which the outcome was measured; ‘year’: the year the outcome study was published; ‘beta1_outcome’: regression coefficient (per additional effect allele) in the outcome study; ‘se_outcome’: standard error of ‘beta1_outcome’; ‘p_outcome’: p-value of ‘beta1_outcome’ and ‘se_outcome’; ‘n_outcome’: number of individuals in the outcome study; ‘n_cases_outcome’: number of cases in the outcome study; ‘n_controls_outcome’: number of controls in the outcome study; ‘n_studies_meta_outcome’: if a meta-analysis, number of studies included; ‘units_outcome’: units of analysis in the outcome study (IVNT stands for inverse normal rank transformed phenotype); ‘dataset’: Phenoscanner dataset ID; ‘beta1_outcome_flipped’: has the sign of ‘beta1_outcome’ been inverted from that provided by Phenoscanner due to calling of the effect vs. non-effect allele? True / False; ‘beta_mr_delta_sec’: beta value using the delta MR method (using up to second order partial derivatives; See the appendix of Lynch and Walsh for further information) using estimates from the secondary cohort; ‘se_mr_delta_sec’: standard error of ‘beta_mr_delta_sec’ using the delta MR method (using up to first order partial derivatives; See the appendix of Lynch and Walsh for further information) using estimates from the secondary cohort; ‘p_mr_delta_sec’: p-value corresponding to ‘beta_mr_delta_sec’ and ‘se_mr_delta_sec’; ‘fdr_sig_mr_delta_sec’: significance of ‘p_mr_delta_sec’ at a False Discovery Rate (FDR) of <5% (True / False).
(TSV)

S7 Table HEIDI and eCAVIAR. Table of the eCAVIAR [44] and HEIDI [23] results for all significant (FDR <0.05) Mendelian Randomization (MR) results using data from GeneAtlas [20]. Columns are ‘snpid’: chromosome_position (GRCh37); ‘exposure’: UniProtID of the protein; ‘hgnc_symbol’: HUGO Gene Nomenclature Committee symbol of the exposure protein; ‘outcome’: outcome code of the UK biobank outcome from GeneAtlas; ‘outcome_description’: description of the UK biobank outcome from GeneAtlas; ‘p_HEIDI’: p-value of the HEIDI statistic; ‘nsnp_HEIDI’: the number of SNPs used in the calculation of the HEIDI statistic; ‘CLPP’: colocalization posterior probability (as per eCAVIAR).
(TSV)

S8 Table ChEMBL results. Compounds targeting the mediators listed in S5 Table. Columns are ‘uniprot’: UniProtID; ‘gene_symbol’: Gene Symbol; ‘target_chembl_id’: CHEMBL ID for this protein; ‘compound_id’: CHEMBL compound ID; ‘max_phase’: CHEMBL-reported maximum phase of drug development for this compound; ‘drug_synonyms’: drug names; ‘indication_class’: CHEMBL-reported indication for this compound.
(TSV)

S9 Table Key of Fig 2A. Key for the abbreviations used in Fig 2A. Columns are ‘Abbreviation’ and ‘Outcome Description’.
(TSV)

S10 Table Key of Fig 2B. Key for the abbreviations used in Fig 2B. Columns are ‘Abbreviation’ and ‘Outcome Description’.
(TSV)
